# Supplementary material for: Does 2x2 airplane passenger contact tracing for infectious respiratory pathogens work? A systematic review of the evidence
Source: PLoS One. 2023 Feb 2;18(2):e0264294. doi: 10.1371/journal.pone.0264294 (PMC9894495; doi:10.1371/journal.pone.0264294)
Supplement: S1 Appendix — (DOCX) [file pone.0264294.s001.docx]

# Supporting information

## S1 Appendix. Search strategy.

**Pubmed and Web of Science search**

¬¬(“communicable diseases” OR “infectious diseases” OR “disease outbreaks” OR “influenza” OR “fomites” OR “coronavirus” OR “coronavirus infections” OR “severe acute respiratory syndrome” OR “respiratory tract infections” OR “middle east respiratory syndrome” OR “common cold”)

AND

("Air travel" OR "inflight" OR "Flight" OR "Aircraft" OR "Air travel" OR “Flying")

AND

(“Pilots” OR “cabin crew” OR “Crew” OR “Passengers”)

**Cochrane search**

(‘communicable diseases’ OR ‘infectious diseases’ OR ‘disease outbreaks’ OR ‘influenza’ OR ‘fomites’ OR ‘coronavirus’ OR ‘coronavirus infections’ OR ‘severe acute respiratory syndrome’ OR ‘respiratory tract infections’ OR ‘middle east respiratory syndrome’ OR ‘common cold’) in Title Abstract Keyword AND ‘Air travel’ OR ‘inflight’ OR ‘Flight’ OR ‘Aircraft’ OR ‘Air travel’ OR ‘Flying’ in Title Abstract Keyword AND ‘Pilots’ OR ‘cabin crew’ OR ‘Crew’ OR ‘Passengers’ in Title Abstract Keyword - (Word variations have been searched)
