# Supplementary material for: Does 2x2 airplane passenger contact tracing for infectious respiratory pathogens work? A systematic review of the evidence
Source: PLoS One. 2023 Feb 2;18(2):e0264294. doi: 10.1371/journal.pone.0264294 (PMC9894495; doi:10.1371/journal.pone.0264294)
Supplement: S2 Appendix — (DOCX) [file pone.0264294.s002.docx]

# Supporting information

## S2 Appendix. Quality assessment tool, adapted from Leitmeyer, et al. [6].

| **Criteria** | **Points awarded or withdrawn** |
| --- | --- |
| **Index case classification** |  |
| Laboratory confirmation | 1 |
| Unspecific clinical presentation or data not provided | 0 |
| **Secondary case ascertainment** |  |
| Laboratory confirmation of all cases | 2 |
| Syndromic (e.g., influenza-like illness) or no comprehensive confirmation of all secondary cases | 1 |
| Not provided | 0 |
| **Contact tracing strategy** |  |
| Comprehensive | 2 |
| Other (two rows, compartment, class, area, retrospective identification) | 0 |
| **Timeliness of contact tracing** |  |
| Within 1 week (1 month for TB) | 2 |
| Within 3 weeks (3 months for TB) | 1 |
| After 3 weeks or more (3 months for TB) | 0 |
| **Completeness of contact tracing: proportion of passengers followed up** |  |
| More than 80% were followed up | 2 |
| Between 80% and 50% were followed up | 1 |
| Less than 50% were followed up or retrospective identification | 0 |
| **Limitations** |  |
| alternative exposure not addressed | -1 |
| 0 −1 Resulting evidence levels: 0–3 low, 4–6 medium, 7–9 high. | |
