# Supplementary material for: Does 2x2 airplane passenger contact tracing for infectious respiratory pathogens work? A systematic review of the evidence
Source: PLoS One. 2023 Feb 2;18(2):e0264294. doi: 10.1371/journal.pone.0264294 (PMC9894495; doi:10.1371/journal.pone.0264294)
Supplement: S1 Table — (DOCX) [file pone.0264294.s003.docx]

# Supporting information

## S1 Table: Supplementary data and evidence assessment.

| Pathogen | Study (First Author and year (investigation #) [reference] | # passengers to be traced | # of passengers traced | # index cases | # 2ndry cases | 2ndry cases within 2 rows | # of people on board | Index Case classification | Secondary case ascertainment | Contact tracing Strategy | Timeliness of contact tracing | Completeness of contact tracing: proportion of passengers followed up | Limitations | Total | Evidence Level |
| --- | --- | --- | --- | --- | --- | --- | --- | --- | --- | --- | --- | --- | --- | --- | --- |
| Diphtheria | Berger, 2016 (1) [56] | 19 | 16 | 1 | 0 | - | - | 1 | 0 | 0 | 2 | 2 | 0 | 5 | Medium |
| H1N1 | Baker, 2010 (1) [12] | 112 | 102 | 12 | 4 | 4 | 379 | 1 | 2 | 0 | 2 | 2 | 0 | 7 | High |
| H1N1 | Foxwell, 2011 (1) [95] | 445 | 145 | 6 | 8 | 8 | 445 | 0 | 2 | 0 | 2 | 0 | 0 | 4 | Medium |
| H1N1 | Foxwell, 2011 (2) [95] | 293 | 131 | 1 | 1 | 1 | 293 | 1 | 2 | 0 | 2 | 0 | 0 | 5 | Medium |
| H1N1 | Han, 2009 (1) [92] | 114 | 114 | 1 | 0 | - | 115 | 1 | 2 | 2 | 0 | 2 | 0 | 7 | High |
| H1N1 | Han, 2009 (2) [92] | 110 | 110 | 1 | 1 | 0 | 111 | 1 | 2 | 2 | 0 | 2 | 0 | 7 | High |
| H1N1 | Han, 2009 (3) [92] | 110 | 110 | 2 | 7 | 1 | 112 | 1 | 2 | 2 | 0 | 2 | 0 | 7 | High |
| H1N1 | Kim, 2010 (1) [89] | 337 | 199 | 1 | 1 | 0 | 338 | 1 | 2 | 2 | 0 | 1 | 0 | 6 | Medium |
| H1N1 | Neatherlin, 2013 (1) [90] | 225 | 146 | 1 | 8 | 3 | 226 | 1 | 1 | 2 | 0 | 1 | 0 | 5 | Medium |
| H1N1 | Neatherlin, 2013 (2) [90] | 167 | 133 | 1 | 4 | 3 | 168 | 1 | 1 | 0 | 0 | 0 | 0 | 2 | Low |
| H1N1 | Ooi, 2010 (1) [88] | 596 | 23 | 1 | 5 | 2 | 596 | 1 | 2 | 2 | 2 | 0 | 0 | 7 | High |
| H1N1 | Pang, 2011 (2) [93] | 1846 | 1846 | 1 | 8 | - | 1854 | 1 | 2 | 2 | 2 | 2 | 0 | 9 | High |
| H1N1 | Pang, 2011, (1) [93] | 1283 | 1283 | 1 | 20 | - | 1303 | 1 | 2 | 2 | 2 | 2 | 0 | 9 | High |
| H1N1 | Young, 2014 & Shankar, 2014 (1) [87, 91] | 278 | 232 | 6 | 6 | 1 | 278 | 1 | 2 | 0 | 2 | 2 | 0 | 7 | High |
| H1N1 | Zhang, 2013 (1) [94] | 274 | 168 | 1 | 9 | - | 274 | 0 | 0 | 2 | 0 | 1 | 0 | 3 | Low |
| ILI | Marsden, 2003 (1) [97] | - | - | 1 | 15 | 9 | - | 0 | 0 | 2 | 2 | 2 | 0 | 6 | Medium |
| Influenza | Moser, 1979 (1) [96] | 54 | 53 | 1 | 38 | - | 54 | 1 | 2 | 0 | 2 | 2 | 0 | 7 | High |
| Measles | Amornkul, 2004 & Lasher, 2004 (1) [57, 78] | 336 | 276 | 1 | 0 | - | 337 | 0 | 0 | 2 | 2 | 2 | 0 | 6 | Medium |
| Measles | Barret, 2018 (1) [58] | - | - | 1 | 1 | - | - | 1 | 2 | 0 | 0 | 0 | 0 | 3 | Low |
| Measles | Barret, 2018 (2) [58] | - | - | 1 | 2 | - | - | 1 | 2 | 0 | 0 | 0 | 0 | 3 | Low |
| Measles | Barret, 2018 (3) [58] | - | - | 1 | 1 | - | - | 1 | 2 | 0 | 0 | 0 | 0 | 3 | Low |
| Measles | Barret, 2018 (4) [58] | - | 250 | 1 | 0 | - | - | 1 | 2 | 0 | 0 | 0 | 0 | 3 | Low |
| Measles | Beard, 2011 (1) [59] | 56 | - | 1 | 4 | 1 | - | 1 | 2 | 0 | 0 | 0 | 0 | 3 | Low |
| Measles | Bitzegeio, 2020 (1) [60] | - | - | 1 | 2 | 0 | - | 1 | 2 | 0 | 0 | 0 | 0 | 3 | Low |
| Measles | Bitzegeio, 2020 (2) [60] | - | 476 | 1 | 3 | - | - | 1 | 2 | 0 | 1 | 0 | 0 | 4 | Medium |
| Measles | CDC, 1983 (1) [62] | - | - | 1 | 1 | - | - | 1 | 2 | 0 | 0 | 0 | 0 | 3 | Low |
| Measles | CDC, 2006 (1) [65] | - | 6 | 1 | 0 | - | - | 1 | 1 | 0 | 0 | 2 | 0 | 4 | Medium |
| Measles | CDC, 2006 (2) [65] | 118 | - | 1 | 0 | - | 118 | 1 | 1 | 0 | 0 | 2 | 0 | 4 | Medium |
| Measles | CDC, 2011 (1) [66] | - | - | 1 | 2 | 0 | - | 1 | 2 | 0 | 1 | 0 | 0 | 4 | Medium |
| Measles | CDC, 2012 (1) [67] | - | - | 1 | 0 | - | - | 1 | 2 | 0 | 1 | 0 | 0 | 4 | Medium |
| Measles | Chen, 2010 (1) [69] | - | - | 1 | 1 | 1 | - | 1 | 2 | 0 | 0 | 0 | 0 | 3 | Low |
| Measles | Chen, 2011 (1) [68] | 145 | 145 | 1 | 0 | - | 145 | 1 | 2 | 2 | 0 | 0 | 0 | 5 | Medium |
| Measles | Cocoros, 2010 (1) [70] | 31 | 29 | 1 | 1 | 1 | - | 1 | 2 | 0 | 2 | 2 | 0 | 7 | High |
| Measles | Coleman, 2009 (1) [71] | 42 | 42 | 1 | 2 | 0 | - | 1 | 2 | 0 | 2 | 2 | 0 | 7 | High |
| Measles | Colier, 2013 (1) [72] | 72 | 32 | 1 | 0 | - | - | 1 | 1 | 1 | 0 | 0 | 0 | 3 | Low |
| Measles | Cotter, 2010 (1) [73] | 321 | 169 | 3 | 2 | 0 | - | 1 | 2 | 2 | 2 | 1 | 0 | 8 | High |
| Measles | Dayan, 2005 & CDC, 2005 &CDC, 2006 (1) [63, 64, 74] | - | - | 117 | 4 | 2 | 10000 | 1 | 0 | 0 | 0 | 0 | 0 | 1 | Low |
| Measles | CDC, 2011 (1) [61] | - | - | 3 | 8 | 3 | - | 1 | 1 | 0 | 0 | 0 | 0 | 2 | Low |
| Measles | Huang, 2018 (1) [76] | - | - | 1 | 2 | 0 | 180 | 1 | 2 | 0 | 0 | 0 | 0 | 3 | Low |
| Measles | Kantele, 2012 (1) [77] | 580 | 580 | 3 | 1 | 0 | 580 | 1 | 2 | 2 | 2 | 0 | 0 | 7 | High |
| Measles | Lu, 2020 (1) [79] | 22 | - | 1 | 1 | - | - | 1 | 2 | 1 | 2 | 2 | 0 | 8 | High |
| Measles | Lu, 2020 (2) [79] | 164 | - | 1 | 0 | - | - | 1 | 2 | 1 | 2 | 2 | 0 | 8 | High |
| Measles | Nelson, 2013 (1) [80] | 3399 | 952 | 74 | 9 | 3 | - | 0 | 0 | 0 | 0 | 0 | 0 | 0 | Low |
| Measles | Ribiero de Barros, 2006 (1) [75] | 334 | 118 | 1 | 2 | 0 | 334 | 1 | 2 | 0 | 0 | 0 | 0 | 3 | Low |
| Measles | Slater, 1995 (1) [81] | - | - | 1 | 8 | - | 350 | 1 | 2 | 0 | 0 | 0 | 0 | 3 | Low |
| Measles | Thole, 2019 (1) [82] | 155 | 155 | 1 | 0 | - | - | 1 | 2 | 2 | 2 | 1 | 0 | 8 | High |
| Measles | Thole, 2019 (2) [82] | - | - | 1 | 2 | - | - | 1 | 2 | 2 | 0 | 0 | 0 | 5 | Medium |
| Measles | van Binnendijk RS, 2008 (1) [83] | - | - | 3 | 3 | - | - | 1 | 2 | 0 | 0 | 0 | 0 | 3 | Low |
| Meningococcal | CDC, 2001, (1) [98] | 2 | 1 | 1 | 0 | - | - | 1 | 0 | 0 | 2 | 1 | 0 | 4 | Medium |
| Meningococcal | O'Connor, 2005 (1) [99] | - | - | 1 | 1 | 0 | - | 1 | 2 | 0 | 0 | 0 | 0 | 3 | Low |
| Meningococcal | Riley, 2006 (1) [100] | - | 200 | 1 | 2 | - | - | 1 | 2 | 0 | 0 | 0 | 0 | 3 | Low |
| MERS | Devi, 2014 (1) [34] | 24 | 21 | 1 | 0 | - | - | 1 | 2 | 0 | 2 | 2 | 0 | 7 | High |
| MERS | Kang, 2015 (1) [29] | 27 | 27 | 1 | 0 | - | - | 1 | 2 | 0 | 2 | 2 | 0 | 7 | High |
| MERS | Kraaij - Dirkzwager, 2014 (1) [30] | 18 | 18 | 2 | 0 | - | - | 1 | 2 | 0 | 2 | 2 | 0 | 7 | High |
| MERS | Kwok-ming, 2015 (1) [31] | - | 42 | 1 | 0 | - | - | 1 | 2 | 0 | 2 | 0 | 0 | 5 | Medium |
| MERS | Mollers, 2015 (1) [32] | 17 | 17 | 2 | 0 | - | - | 1 | 2 | 0 | 2 | 2 | 0 | 7 | High |
| MERS | Parry-Ford, 2015 & Lippold, 2017 (1) [27, 28] | 173 | 154 | 1 | 0 | - | - | 0.75 | 2 | 1 | 0.75 | 0.5 | 0 | 5 | Medium |
| MERS | Parry-Ford, 2015 & Lippold, 2017 (2) [27, 28] | 574 | 541 | 1 | 0 | - | - | 1 | 2 | 1 | 0.75 | 1.25 | 0 | 6 | Medium |
| MERS | Plipat, 2015 (1) [33] | 26 | 26 | 1 | 0 | - | - | 1 | 2 | 0 | 2 | 2 | 0 | 7 | High |
| MERS | Puzelli, 2013 (1) [35] | 9 | 9 | 1 | 0 | - | - | 1 | 2 | 0 | 2 | 2 | 0 | 7 | High |
| MERS | Racelis, 2015 (1) [36] | 237 | 85 | 1 | 0 | - | - | 1 | 2 | 0 | 2 | 0 | 0 | 5 | Medium |
| MERS | Tsiodras, 2014 (1) [37] | - | 12 | 1 | 0 | - | - | 1 | 2 | 0 | 2 | 0 | 0 | 5 | Medium |
| MERS | Wu, 2015 (1) [38] | 6 | 6 | 1 | 0 | - | - | 1 | 2 | 0 | 2 | 2 | 0 | 7 | High |
| Mumps | CDC, 2006 (1) [85] | 575 | 132 | 11 | 2 | - | - | 1 | 2 | 0 | 0 | 0 | 0 | 3 | Low |
| Mumps | Nelson, 2012 (1) [84] | 166 | 21 | 2 | 0 | - | - | 1 | 0 | 0 | 0 | 0 | 0 | 1 | Low |
| Rubella | Kim, 2012 (1) [86] | 250 | 215 | 1 | 0 | - | - | 1 | 0 | 0 | 0 | 2 | 0 | 3 | Low |
| SARS | Breugelmans, 2010 (1) [24] | 250 | 36 | 1 | 0 | - | - | 1 | 0 | 0 | 2 | 2 | -1 | 4 | Medium |
| SARS | Desenclos, 2004 (1) [25] | 7 | 7 | 1 | 2 | 1 | 402 | 1 | 2 | 0 | 1 | 2 | 0 | 6 | Medium |
| SARS | Olsen, 2003 (1) [8] | 315 | 74 | 1 | 0 | - | 315 | 1 | 0 | 2 | 0 | 0 | 0 | 3 | Low |
| SARS | Olsen, 2003 (2) [8] | 120 | 65 | 1 | 22 | 5 | 120 | 1 | 1 | 2 | 0 | 1 | 0 | 5 | Medium |
| SARS | Olsen, 2003 (3) [8] | 246 | 166 | 4 | 1 | - | 246 | 1 | 0 | 2 | 1 | 1 | 0 | 5 | Medium |
| SARS | Vogt, 2006 (1) [23] | 334 | 108 | 1 | 0 | - | 334 | 1 | 2 | 2 | 0 | 0 | -1 | 4 | Medium |
| SARS | Vogt, 2006 (2) [23] | 296 | 46 | 1 | 0 | - | 296 | 1 | 2 | 2 | 0 | 0 | -1 | 4 | Medium |
| SARS | Vogt, 2006 (3) [23] | 374 | 47 | 1 | 0 | - | 374 | 1 | 2 | 2 | 0 | 0 | -1 | 4 | Medium |
| SARS | Vogt, 2006 (4) [23] | 133 | 73 | 1 | 0 | - | 133 | 1 | 2 | 2 | 0 | 1 | -1 | 5 | Medium |
| SARS | Vogt, 2006 (5) [23] | 212 | 25 | 1 | 0 | - | 212 | 1 | 2 | 2 | 0 | 0 | -1 | 4 | Medium |
| SARS | Vogt, 2006 (6) [23] | 32 | 8 | 1 | 0 | - | 32 | 1 | 2 | 2 | 0 | 0 | -1 | 4 | Medium |
| SARS | Vogt, 2006 (7) [23] | 385 | 32 | 1 | 0 | - | 385 | 1 | 2 | 2 | 0 | 0 | -1 | 4 | Medium |
| SARS | Wilder-Smith, 2003 (1) [26] | - | - | 3 | 0 | - | - | 0 | 2 | 0 | 2 | 0 | 0 | 4 | Medium |
| SARS | Wilder-Smith, 2003 (2) [26] | - | - | 1 | 1 | 0 | - | 0 | 2 | 0 | 2 | 0 | 0 | 4 | Medium |
| SARS | Wilder-Smith, 2003 (3) [26] | - | - | 1 | 0 | - | - | 0 | 2 | 0 | 2 | 0 | 0 | 4 | Medium |
| SARS | Wilder-Smith, 2003 (4) [26] | - | - | 1 | 0 | - | - | 0 | 2 | 0 | 2 | 0 | 0 | 4 | Medium |
| SARS | Wilder-Smith, 2003 (5) [26] | - | - | 1 | 0 | - | - | 0 | 2 | 0 | 2 | 0 | 0 | 4 | Medium |
| SARS | Wilder-Smith, 2003 (6) [26] | - | - | 1 | 0 | - | - | 0 | 2 | 0 | 2 | 2 | 0 | 6 | Medium |
| SARS | Wilder-Smith, 2003 (7) [26] | - | - | 1 | 0 | - | - | 0 | 2 | 0 | 2 | 0 | 0 | 4 | Medium |
| SARS-CoV-2 | Bae, 2020 (1) [103] | 287 | 287 | 6 | 1 | 0 | 299 | 1 | 2 | 2 | 2 | 2 | -1 | 8 | High |
| SARS-CoV-2 | Bae, 2020 (2) [103] | 202 | 202 | 3 | 1 | - | 205 | 1 | 2 | 2 | 2 | 2 | -1 | 8 | High |
| SARS-CoV-2 | Bernard Stoecklin, 2020 (1) [104] | 13 | - | 1 | 0 | - | 234 | 1 | 2 | 0 | 1 | 0 | 0 | 4 | Medium |
| SARS-CoV-2 | Blomquist, 2021 (1) [105] | 425 | 79 | 55 | 5 | 4 | 2368 | 1 | 2 | 0 | 2 | 2 | 0 | 7 | High |
| SARS-CoV-2 | Bohmer, 2020 (1) [106] | - | - | 1 | 0 | - | - | 1 | 2 | 0 | 0 | 0 | -1 | 2 | Low |
| SARS-CoV-2 | Bohmer, 2020 (2) [106] | - | - | 1 | 0 | - | - | 1 | 2 | 2 | 2 | 2 | 0 | 9 | High |
| SARS-CoV-2 | Burke, 2020 (1) [102] | 13 | 13 | 1 | 0 | - | - | 1 | 2 | 0 | 2 | 2 | 0 | 7 | High |
| SARS-CoV-2 | Chen 2020 (1) [107] | 330 | 330 | 11 | 1 | 1 | 342 | 1 | 2 | 2 | 2 | 1 | 0 | 8 | High |
| SARS-CoV-2 | Choi, 2020 (2) [108] | 294 | 0 | 2 | 2 | - | 294 | 1 | 2 | 2 | 0 | 2 | 0 | 7 | High |
| SARS-CoV-2 | Draper, 2020 (1) [109] | 389 | 326 | 14 | 0 | - | - | 1 | 2 | 2 | 1 | 2 | 0 | 8 | High |
| SARS-CoV-2 | Eichler, 2021 (1) [101] | 148 | 148 | 1 | 2 | 2 | 149 | 1 | 2 | 0 | 2 | 2 | 0 | 7 | High |
| SARS-CoV-2 | Eichler, 2021 (2) [101] | - | - | 2 | 1 | 1 | 94 | 1 | 2 | 2 | 2 | 2 | 0 | 9 | High |
| SARS-CoV-2 | Eldin, 2020 (1) [115] | - | - | 1 | 1 | - | - | 1 | 2 | 0 | 2 | 0 | 0 | 5 | Medium |
| SARS-CoV-2 | Eldin, 2020 (2) [115] | - | - | 1 | 0 | - | - | 1 | 2 | 0 | 2 | 2 | 0 | 7 | High |
| SARS-CoV-2 | Hoehl, 2020 (1) [110] | 95 | 95 | 7 | 2 | 2 | 102 | 1 | 2 | 2 | 1 | 0 | 0 | 6 | Medium |
| SARS-CoV-2 | Khanh, 2020 (1) [111] | 216 | 184 | 1 | 15 | 11 | 217 | 1 | 2 | 2 | 2 | 2 | 0 | 9 | High |
| SARS-CoV-2 | Murphy, 2020 (1) [3] | 60 | 48 | 1 | 13 | - | 61 | 1 | 2 | 2 | 2 | 2 | 0 | 9 | High |
| SARS-CoV-2 | Nir-Paz, 2020 (1) [116] | 9 | 9 | 2 | 0 | - | 11 | 1 | 1 | 0 | 0 | 0 | 0 | 2 | Low |
| SARS-CoV-2 | Nye, 2021 (1) [117] | - | - | 1 | 3 | 3 | - | 1 | 2 | 0 | 0 | 2 | 0 | 5 | Medium |
| SARS-CoV-2 | Nye, 2021 (2) [117] | - | - | 2 | 2 | 2 | - | 1 | 2 | 0 | 0 | 2 | 0 | 5 | Medium |
| SARS-CoV-2 | Nye, 2021 (3) [117] | - | - | 3 | 1 | 1 | - | 1 | 2 | 0 | 0 | 2 | 0 | 5 | Medium |
| SARS-CoV-2 | Nye, 2021 (4) [117] | - | - | 6 | 1 | 0 | - | 1 | 2 | 0 | 0 | 2 | 0 | 5 | Medium |
| SARS-CoV-2 | Nye, 2021 (5) [117] | - | - | 40 | 3 | - | - | 1 | 2 | 0 | 0 | 2 | 0 | 5 | Medium |
| SARS-CoV-2 | Nye, 2021 (6) [117] | - | - | 5 | 3 | 3 | - | 1 | 2 | 0 | 0 | 2 | 0 | 5 | Medium |
| SARS-CoV-2 | Pavli, 2020 (1) [112] | - | 981 | 21 | 5 | 4 | 2334 | 0 | 0 | 0 | 0 | 0 | 0 | 0 | Low |
| SARS-CoV-2 | Qian, 2020 (1) [113] | - | - | 1 | 10 | - | - | 1 | 0 | 0 | 0 | 0 | 0 | 1 | Low |
| SARS-CoV-2 | Schwartz, 2020 (1) [114] | 25 | 25 | 1 | 0 | - | 350 | 1 | 2 | 0 | 0 | 0 | 0 | 3 | Low |
| SARS-CoV-2 | Speake, 2020 (1) [10] | 241 | - | 11 | 11 | 8 | 241 | 1 | 2 | 1 | 2 | 2 | 0 | 8 | High |
| SARS-CoV-2 | Swadi, 2020 (1) [1] | 84 | 84 | 2 | 4 | 4 | 86 | 1 | 2 | 0 | 0 | 0 | 0 | 3 | Low |
| TB | Abubakar, 2008 (1) [42] | 28 | 3 | 1 | 0 | - | - | 0 | 2 | 0 | 0 | 2 | -1 | 3 | Low |
| TB | Abubakar, 2008 (2) [42] | 28 | 3 | 1 | 0 | - | - | 0 | 2 | 0 | 0 | 0 | -1 | 1 | Low |
| TB | Abubakar, 2008 (3) [42] | 22 | 7 | 1 | 0 | - | - | 0 | 2 | 0 | 0 | 0 | -1 | 1 | Low |
| TB | Abubakar, 2008 (4) [42] | 32 | 4 | 1 | 0 | - | - | 1 | 2 | 0 | 0 | 0 | -1 | 2 | Low |
| TB | Abubakar, 2008 (5) [42] | - | 4 | 1 | 0 | - | - | 1 | 2 | 0 | 0 | 0 | -1 | 2 | Low |
| TB | Abubakar, 2008 (6) [42] | - | 2 | 1 | 0 | - | - | 1 | 2 | 0 | 0 | 0 | -1 | 2 | Low |
| TB | Abubakar, 2008 (7) [42] | - | - | 1 | 0 | - | - | 1 | 2 | 0 | 0 | 0 | -1 | 2 | Low |
| TB | Abubakar, 2008 (8) [42] | 41 | 8 | 1 | 0 | - | - | 1 | 2 | 0 | 0 | 0 | -1 | 2 | Low |
| TB | Abubakar, 2008 (9) [42] | 43 | 15 | 1 | 0 | - | - | 1 | 2 | 0 | 0 | 0 | -1 | 2 | Low |
| TB | Abubakar, 2008 (10) [42] | 47 | 7 | 1 | 0 | - | - | 1 | 2 | 0 | 0 | 0 | -1 | 2 | Low |
| TB | Abubakar, 2008 (11) [42] | - | - | 1 | 0 | - | - | 1 | 2 | 0 | 0 | 0 | -1 | 2 | Low |
| TB | an der Heiden, 2017 (1) [43] | 162 | 135 | 1 | 1 | 0 | 163 | 1 | 2 | 0 | 2 | 2 | 0 | 7 | High |
| TB | Beller, 1996 (1) [40] | 12 | 12 | 1 | 0 | - | 13 | 1 | 2 | 2 | 2 | 2 | 0 | 9 | High |
| TB | CDC, 1995 (1) [39] | 274 | 266 | 1 | 6 | - | - | 1 | 2 | 0 | 0 | 2 | 0 | 5 | Medium |
| TB | CDC, 1995 (2) [39] | 343 | 79 | 1 | 0 | - | 344 | 1 | 2 | 2 | 0 | 0 | 0 | 5 | Medium |
| TB | CDC, 1995 (3) [39] | 92 | 22 | 1 | 0 | - | 93 | 1 | 2 | 2 | 0 | 0 | 0 | 5 | Medium |
| TB | CDC, 1995 (4) [39] | 219 | 142 | 1 | 0 | - | 220 | 1 | 2 | 2 | 0 | 1 | 0 | 6 | Medium |
| TB | CDC, 1995 (5) [39] | 661 | 87 | 1 | 0 | - | 662 | 1 | 2 | 2 | 0 | 0 | 0 | 5 | Medium |
| TB | CDC, 1995 (6) [39] | 925 | 802 | 1 | 4 | - | 926 | 1 | 2 | 2 | 0 | 2 | 0 | 7 | High |
| TB | CDC, 2012 (1) [41] | 15 | 2 | 1 | 0 | - | - | 1 | 2 | 0 | 0 | 0 | 0 | 3 | Low |
| TB | CDC, 2012 (2) [41] | - | 15650 | 390 | 0 | - | - | 1 | 2 | 0 | 0 | 0 | 0 | 3 | Low |
| TB | Chemardin, 2007 (1) [44] | 11 | 7 | 1 | 0 | - | - | 1 | 0 | 2 | 0 | 1 | -1 | 3 | Low |
| TB | Driver, 1994 (1) [45] | 339 | 334 | 1 | 9 | - | - | 1 | 2 | 0 | 2 | 2 | 0 | 7 | High |
| TB | Flanagan, 2016 (1) [46] | 20 | 20 | 1 | 0 | - | - | 1 | 2 | 0 | 1 | 2 | 0 | 6 | Medium |
| TB | Flanagan, 2016 (2) [46] | 24 | 22 | 1 | 0 | - | - | 1 | 2 | 0 | 1 | 2 | 0 | 6 | Medium |
| TB | Flanagan, 2016 (3) [46] | 15 | 6 | 1 | 0 | - | - | 1 | 2 | 0 | 1 | 0 | 0 | 4 | Medium |
| TB | Flanagan, 2016 (4) [46] | 39 | 39 | 1 | 0 | - | - | 1 | 2 | 0 | 2 | 2 | 0 | 7 | High |
| TB | Flanagan, 2016 (5) [46] | 27 | 18 | 1 | 0 | - | - | 1 | 2 | 0 | 1 | 1 | 0 | 5 | Medium |
| TB | Flanagan, 2016 (6) [46] | 10 | 7 | 1 | 0 | - | - | 1 | 2 | 0 | 2 | 1 | 0 | 6 | Medium |
| TB | Flanagan, 2016 (7) [46] | 27 | 16 | 1 | 0 | - | - | 1 | 2 | 0 | 1 | 1 | 0 | 5 | Medium |
| TB | Flanagan, 2016 (8) [46] | 26 | 26 | 1 | 0 | - | - | 1 | 2 | 0 | 2 | 2 | 0 | 7 | High |
| TB | Flanagan, 2016 (9) [46] | 44 | 44 | 1 | 0 | - | - | 1 | 2 | 0 | 0 | 2 | 0 | 5 | Medium |
| TB | Kenyon, 1996 (1) [47] | - | 298 | 1 | 0 | - | - | 1 | 2 | 2 | 2 | 0 | -1 | 6 | Medium |
| TB | Kenyon, 1996 (2) [47] | - | 104 | 1 | 0 | - | - | 1 | 2 | 2 | 0 | 1 | 0 | 6 | Medium |
| TB | Kenyon, 1996 (3) [47] | - | 109 | 1 | 0 | - | - | 1 | 2 | 2 | 0 | 1 | 0 | 6 | Medium |
| TB | Kenyon, 1996 (4) [47] | - | 249 | 1 | 6 | 4 | - | 1 | 2 | 2 | 0 | 1 | 0 | 6 | Medium |
| TB | Kornylo-Duong, 2010 (1) [48] | 35 | 22 | 1 | 0 | - | - | 1 | 2 | 0 | 0 | 1 | 0 | 4 | Medium |
| TB | Kornylo-Duong, 2010 (2) [48] | 42 | 29 | 1 | 0 | - | - | 1 | 2 | 0 | 0 | 1 | 0 | 4 | Medium |
| TB | Kornylo-Duong, 2010 (3) [48] | 25 | 11 | 1 | 0 | - | - | 1 | 2 | 0 | 0 | 0 | 0 | 3 | Low |
| TB | Kornylo-Duong, 2010 (4) [48] | 29 | 17 | 1 | 0 | - | - | 1 | 2 | 0 | 0 | 1 | 0 | 4 | Medium |
| TB | Marienau, 2010 (1) [49] | 4450 | 861 | 131 | 0 | - | - | 1 | 2 | 0 | 0 | 0 | 0 | 3 | Low |
| TB | Mcfarland, 1993 (1) [50] | 343 | 136 | 1 | 0 | - | 344 | 1 | 1 | 2 | 2 | 0 | 0 | 6 | Medium |
| TB | Miller, 1996 (1) [51] | 219 | 120 | 1 | 2 | - | - | 1 | 2 | 2 | 0 | 1 | 0 | 6 | Medium |
| TB | Moore, 1996 (1) [20] | 161 | 120 | 1 | 0 | - | - | 0 | 0 | 0 | 0 | 0 | -1 | -1 | Low |
| TB | Parmet, 1999 (1) [55] | 48 | 48 | 1 | 0 | - | - | 1 | 2 | 0 | 0 | 2 | 0 | 5 | Medium |
| TB | Scholten, 2008 (1) [21] | - | 2472 | 98 | 0 | - | - | 1 | 2 | 0 | 0 | 0 | 0 | 3 | Low |
| TB | Thibealut, 2012 (1) [52] | 56 | 32 | 1 | 0 | - | - | 1 | 2 | 0 | 1 | 1 | 0 | 5 | Medium |
| TB | Vassiloyanakopoulos, 1999 (1) [53] | 147 | 24 | 1 | 0 | - | 148 | 1 | 2 | 2 | 2 | 0 | 0 | 7 | High |
| TB | Wang, 1998 (1) [118] | 308 | 277 | 1 | 3 | 0 | 309 | 1 | 2 | 2 | 2 | 2 | 0 | 9 | High |
| TB | Whitlock, 2001 (1) [54] | - | 67 | 1 | 0 | - | - | 1 | 2 | 0 | 0 | 2 | 0 | 5 | Medium |
| TB | Whitlock, 2001 (2) [54] | - | 171 | 1 | 0 | - | - | 1 | 2 | 2 | 0 | 2 | 0 | 7 | High |
