# Supplementary figures and images for: Does 2x2 airplane passenger contact tracing for infectious respiratory pathogens work? A systematic review of the evidence
Source: PLoS One. 2023 Feb 2;18(2):e0264294. doi: 10.1371/journal.pone.0264294 (PMC9894495; doi:10.1371/journal.pone.0264294)

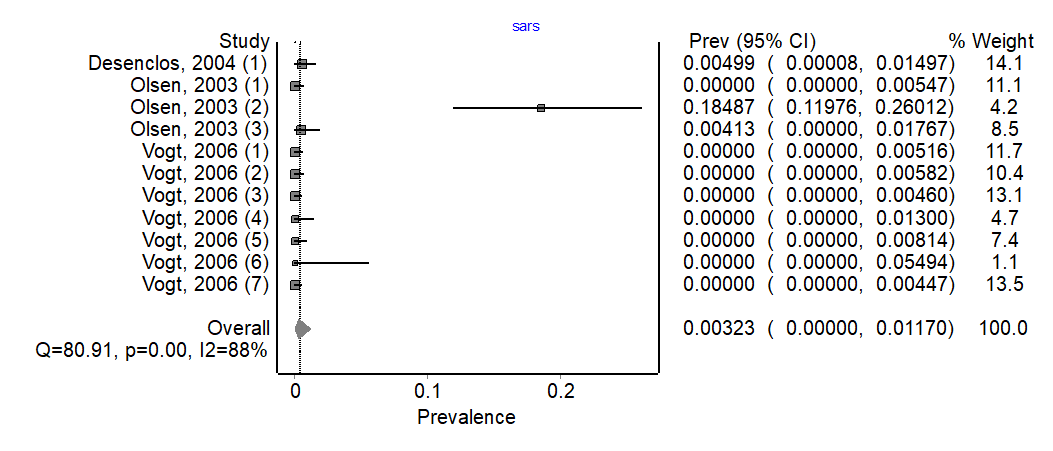

Supplement: S1 Fig — (TIF) [file pone.0264294.s004.tif]

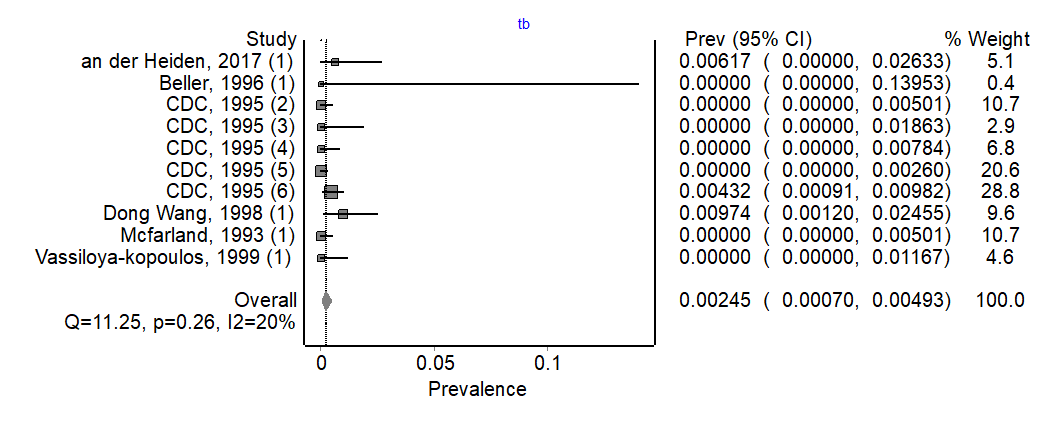

Supplement: S2 Fig — (TIF) [file pone.0264294.s005.tif]

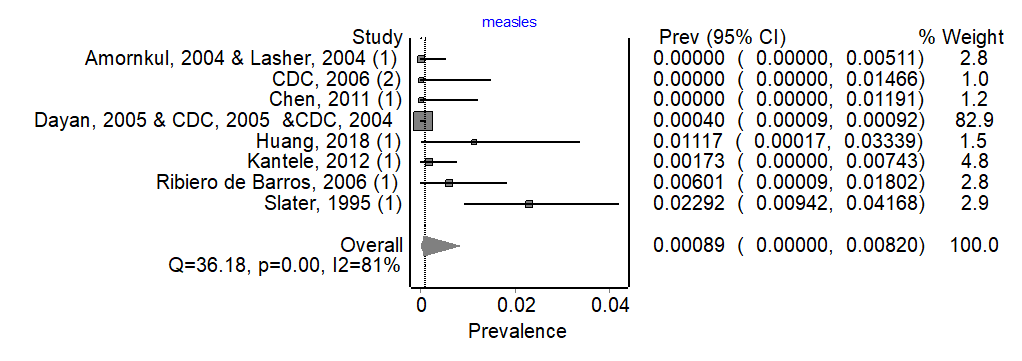

Supplement: S3 Fig — (TIF) [file pone.0264294.s006.tif]
