# Supplementary material for: Does 2x2 airplane passenger contact tracing for infectious respiratory pathogens work? A systematic review of the evidence
Source: PLoS One. 2023 Feb 2;18(2):e0264294. doi: 10.1371/journal.pone.0264294 (PMC9894495; doi:10.1371/journal.pone.0264294)
Supplement: Attachment — Submitted filename: Rafferty Response to reviewers_PLOS ONE_R2.docx [file pone.0264294.s010.docx]

## Response to reviewers

Reviewer #2: Thank you for the thorough revision. Nevertheless, I have 2 minimal comments:

1) Maybe I don't have the right version of the revised manuscript, but the reference to my comment #6 is still missing (please see p. 7, line 137-142).

*Thanks very much. We have inserted the following two references that discuss contact tracing across multiple flights to address this comment:*

1. *Moore M, Fleming KS, Sands L. A passenger with pulmonary/laryngeal tuberculosis: No evidence of transmission on two short flights. Aviat Space Environ Med. 1996;67(11):1097-100.*
2. *Scholten D, Saunders A, Dawson K, Wong T, Ellis E. Air travel by individuals with active tuberculosis: reporting patterns and epidemiologic characteristics, Canada 2006-2008. Travel Med Infect Dis. 2010;8(2):113-9. doi: 10.1016/j.tmaid.2010.02.002.*

2) Line 288-289: "In addition, it is likely that the many instances where infectious cases transmitted to one no other passengers were not published." If necessary, the author could check this sentence again, as it is still not entirely clear. Perhaps an "or" is missing.

*Thanks very much. This has been corrected.*

Thank you for your efforts.
